# Cognitive Performance Among Older Adults with Subjective Cognitive Decline

**DOI:** 10.3390/geriatrics10020039

**Published:** 2025-03-13

**Authors:** Ramón López-Higes, Susana Rubio-Valdehita, David López-Sanz, Sara M. Fernandes, Pedro F. S. Rodrigues, María Luisa Delgado-Losada

**Affiliations:** 1 Departamento de Psicología Experimental, Complutense University of Madrid (UCM), 28223 Madrid, Spain; davidlopezsanz@ucm.es (D.L.-S.); mldelgado@ucm.es (M.L.D.-L.); 2 Departamento de Psicología Social, del Trabajo y Diferencial, Complutense University of Madrid (UCM), 28223 Madrid, Spain; srubiova@ucm.es; 3 Centro de Neurociencia Cognitiva y Computacional (C3N), Universidad Complutense de Madrid, 28015 Madrid, Spain; 4 CINTESIS.UPT@RISE-Health, Portucalense University, 4200-072 Porto, Portugal; sarab@upt.pt (S.M.F.); prodrigues@upt.pt (P.F.S.R.)

**Keywords:** subjective cognitive decline, healthy older adults, memory, executive functions, language

## Abstract

**Objectives:** The main objective of this cross-sectional study was to investigate if there are significant differences in cognition between a group of older adults with subjective cognitive decline (SCD) and cognitively intact controls. **Methods:** An initial sample of 132 older people underwent an extensive neuropsychological evaluation (memory, executive functions, and language) and were classified according to diagnostic criteria. Two groups of 33 subjects each, controls and SCD, were formed using an a priori case-matching procedures in different variables: age, biological sex, years of education, cognitive reserve, and Mini-Mental State Exam. **Results:** The mean age and standard deviation in the control group were equal to 70.39 ± 4.31 years, while in the SCD group, they were 70.30 ± 4.33 years. The number of males (*n* = 9) and females (*n* = 24) was equal in both groups; the means of years of education were also quite similar. SCD participants have a significantly lower mood than the controls. Significant differences between groups were obtained in delayed recall, inhibitory control, and comprehension of sentences not fitted to canonical word order in Spanish. A logistic regression revealed that a lower score on the Stroop’s interference condition is associated with a higher likelihood of having SCD. Finally, ROC analysis provided a model that performs better than random chance, and a cut-off score in Stroop’s interference condition equal to 49 was suggested for clinically differentiating the two groups. **Conclusions:** This study highlights that, compared to a matched control group, participants with SCD showed subtle but significant neuropsychological differences.

## 1. Introduction

The subjective experience of cognitive decline without objective evidence of cognitive deficits was referred to as subjective cognitive decline (SCD) by Jessen et al. [1]. However, there is no accepted gold standard definition of SCD (see alternative proposals from Pettersen et al., or Jessen et al. [2,3]). It is usually considered that these individuals show cognitive performance within the normal range in standardized cognitive tests and the absence of major psychiatric, neurological, or medical disorders affecting cognition.

SCD in older adults is understood as an early marker of cognitive impairment or neurodegeneration like Alzheimer’s Disease (AD) [4,5] since many reports have indicated that it is associated with an increased risk of clinical progression to mild cognitive impairment (MCI) in cognitively normal individuals [6,7]. SCD detection allows for early intervention and treatment; thus, it holds significant importance [8]. Furthermore, SCD can vary over time; that is, while some individuals show stability in their cognitive state, others may exhibit fluctuations, progression [9,10], or even reversion [11]. These features highlight the need for careful monitoring and assessment over time.

Several socio-demographic features are found among people with SCD. For instance, women are more likely to have SCD than men [12]. People with SCD are younger and have a higher education than patients with MCI and AD dementia [13]. Hypertension, smoking, less physical activity, and depression are also associated with the increased occurrence of SCD [4]. Workers who have cognitively demanding occupations are more sensitive to the changes in cognitive decline and thus report cognitive complaints more frequently [14].

Research has revealed both significant correlations and small discrepancies between older adults’ self-perceived cognitive abilities and their objective cognitive performance. A systematic review and meta-analysis of 50 studies showed a small but significant relationship between subjective and objective cognitive functioning in older adults [8]. A more recent systematic review [15] has shown that SCD was associated with poorer objective cognitive performance on measures of global cognition and memory longitudinally compared to non-SCD status, but this association was inconsistent in cross-sectional studies.

Personality traits like extraversion and agreeableness can moderate the relationship between subjective and objective cognition in aging adults, potentially influencing how individuals perceive and perform in cognitive tasks [16]. Furthermore, subjective cognitive complaints can have significant implications for an individual’s quality of life, regardless of objective cognitive performance [17]. In turn, anxiety or depression may lead to an increase in subjective cognitive complaints [18]. This suggests that subjective complaints can sometimes be an early indicator of underlying cognitive changes that may not easily be fully captured by objective tests [19].

Although individuals with subjective cognitive decline (SCD) may not show clinically significant cognitive impairments, increasing evidence suggests that there are subtle cognitive and neurobiological differences when compared to age-matched healthy older adults. These differences can be detected through specialized assessments [20]. This is the primary focus of the current study.

Concerning memory, research has indicated that older adults with SCD may exhibit slight alterations in working memory compared to their cognitively healthy peers [21]. For example, a significant relationship between amyloid burden and verbal memory decline has been found in individuals with SCD over two years [22]. Given that executive functions play a crucial role in various cognitive processes, it is also important to consider potential differences between SCD patients and healthy older adults. Studies have shown that lower scores in episodic memory and executive function are associated with higher levels of SCD, indicating potential differences in cognitive functioning between the two groups [23]. Additionally, research suggests that executive skills, which include abilities related to planning, organization, and cognitive flexibility, may play a significant role in language processing in individuals with SCD [24]. This study showed differences in lexical retrieval and sentence comprehension between individuals with SCD and healthy controls, indicating potential disparities in linguistic performance related to executive functions. A recent study [25] highlights that discrepancy scores in the language domain play a role in distinguishing individuals with SCD from those with normal cognition.

The primary aim of this study was to assess whether significant differences exist in the performance of older adults with SCD across various cognitive domains (memory, executive functions, and language) compared to a control group of cognitively intact older adults, matched for age, biological sex, years of education, cognitive reserve, and general cognitive status. Finally, the second goal was to determine which variable(s), if any, would have a significant role in subjects’ classification. Regarding the first objective, we hypothesize that there will be differences between the groups in executive functions since these play an important role in different cognitive processes. Secondly, we expect that dependent measures related to executive functions would play a prominent role in participants’ classification into SCD or normal category.

## 2. Materials and Methods

### 2.1. Participants

One hundred and thirty-two Spanish-speaking European older adults (between 65 and 85 years old) voluntarily participated in a study focused on subjective cognitive decline characterization and healthy control follow-up. All participants met the following criteria: (1) Mini-Mental State Examination (MMSE; Spanish adaptation by Lobo et al. [26]) score greater or equal to 27, indicating normal cognitive functioning; (2) Yesavage Geriatric Depression Scale (GDS-15; [27]) score lower than 5 points, indicating the absence of depression; (3) normal performance on the Logical Memory (LM) delayed recall subtest of the Wechsler Memory Scale-Third Edition (WMS-III; [28]), to exclude memory impairment. All participants had normal or corrected hearing and vision. Participants come from centers and associations of older adults in the city of Madrid (Spain).

Using a priori method of matching individuals by age, gender, years of education, cognitive reserve, and MMSE score, two groups were finally composed. Half of the selected participants were identified as older adults with SCD (*n*_1_ = 33; 9 males and 24 females) according to Jessen et al.’s criteria [1]. These inclusion criteria for individuals with SCD include: (1) a self-experienced persistent decline in the individual cognitive capacity in comparison to previous normal status and being aware of these changes; (2) subjective concerns expressed about cognitive decline; (3) the onset within the last five years; (4) feelings of a worse performance compared to peers. The control group (*n*_2_ = 33; 9 males and 24 females) did not present subjective complaints within any cognitive domain.

All participants were informed about the objectives of the study and were invited to participate after signing an informed consent form. The present study complied with the ethical standards of the Declaration of Helsinki and was approved by the Ethical Committee of the San Carlos Clinical Hospital in Madrid (internal code: 18/422-E_BS, approval date: 17 December 2018).

### 2.2. Instruments

A brief questionnaire was applied to all participants to obtain data of interest for the study, such as age and the number of years of education. We used the Cognitive Reserve Questionnaire (CRQ; Cuestionario de Reserva Cognitiva; [29]) for CR estimation. The CRQ contains 8 items that measure various aspects of the subject’s activity: schooling, completion of courses, parental schooling, occupational history, musical training, and proficiency in other languages. The CRQ also asks about the frequency with which activities, such as reading and playing intellectual games, have been carried out. The maximum score sum for the CRQ is equal to 25 points.

As mentioned in the Participants subsection, three tests were also used for screening purposes: MMSE, GDS-15, and the Logical Memory (LM) delayed recall subtest of the WMS-III.

Episodic memory was assessed with the Weschler Memory Scale III (WMS-III) Word List subtest (Spanish version; [28]). To assess a person’s working memory and short-term memory capacity, the WMS-III Digit Span subtest was used, which evaluates both forward and backward digit span. The assessment protocol also included a digit reordering task [30]. While the backward digits task assesses working memory and information manipulation skills (rearranging digits in the opposite direction), the digit reordering task asks the participant to rearrange digits according to a specific rule, which can be in ascending or descending order, or following another specified criterion; therefore, this task also assesses working memory, but focuses more on the ability to apply rules and perform rearrangement based on specific criteria.

Executive function processes, such as inhibitory control and processing speed, were assessed by the Stroop test [31] and the Trail Making Test Part A (TMT-A; [32]), respectively. The words in the Stroop test were augmented in size to avoid perceptual problems in participants.

Regarding the language domain, the 60-item Boston Naming Test (BNT; Adapted Spanish edition [33]) was used to explore naming. Sentence comprehension was assessed using a sentence–picture verification task, included in the ECCO_Senior test (Exploración Cognitiva de la Comprensión de Oraciones para mayores; English translation: Cognitive Assessment of Sentence Comprehension for seniors; [34]). The canonical word order in Spanish is Subject + Verb + Object (it is an SVO language). Passive structures are a good example of non-canonical word order sentences. A subset of syntactically complex sentences from the original ECCO_Senior test was selected for the study: non-canonical sentences and sentences containing two propositions (or verbs).

### 2.3. Procedure

The tests mentioned here were used in the context of a larger research project on aging. During the first session, an experienced neuropsychologist conducted the initial structured interview. The screening tests (MMSE, GDS-15, and WMS-III Logical Memory subtest) were also applied. The rest of the tests were applied in the following two sessions, along with other neuropsychological tests and questionnaires selected for the broader study, in the same order for all participants. All the neuropsychological tests were administered following the instructions in their respective manuals.

### 2.4. Data Analysis

First, descriptive statistics are shown for the variables used to match the subjects in both groups, as well as for all dependent measures. Multivariate ANOVAs were used to study differences between SCD and control participants. Partial eta squared was selected as an estimation of effect size; observed power was also included.

Logistic regression was used to classify the participants using as predictors all the significant variables in previous multivariate ANOVAs. A final ROC curve analysis was also performed with the variables that had an important role in subjects’ classification to obtain cut-off values with clinical significance.

## 3. Results

Group mean ages are almost the same (70.39 vs. 70.30 years old), the percentage of females and males was the same in both groups (72% and 38%, respectively), and years of education range from medium to high level (14 ± 5 years). Cognitive reserve means are quite similar when the two groups are compared (15 points from a maximum of 25), and MMSE means denote normal cognitive state in both groups (higher of 28 points). The SCD group has a higher GDS-15 score than the control group, denoting lower mood but not depression. Means and standard deviations corresponding to all variables used to match subjects and GDS-15 scores are shown in Table 1.

A first multivariate ANOVA showed that there are no significant differences between groups in age, years of education, cognitive reserve, and MMSE. Another multivariate ANOVA with all dependent measures across cognitive domains (memory, executive functions, and language) reveals that the two groups significantly differ in the Word List delayed recall, the Stroop’s interference condition, and in the comprehension of sentences not fitted to canonical word order in Spanish (ECCO test). See also the descriptives in Table 1. The right side of Table 1 shows the *F*-statistic value, partial eta square, and observed power in all dependent variables.

### 3.1. Subjects’ Classification

Logistic regression results pointed out that only Stroop’s interference condition reached the criteria to be included in the final equation, and it correctly classified 71.2% of cases. It should be noted that Delayed Recall approaches statistical significance in the analysis. Table 2 summarizes the results obtained in this analysis.

A negative beta suggests that lower scores in the Stroop’s interference condition are associated with better subject classification into the SCD category. Exp(B) represents the odds ratio associated with a one-unit change in the predictor variable. In this case, Exp(B) suggests that for every one-unit increase in the predictor variable, the odds of the event happening (correct classification in the SCD group) are approximately 0.914 times lower.

### 3.2. ROC Analysis

**Figure 1:**
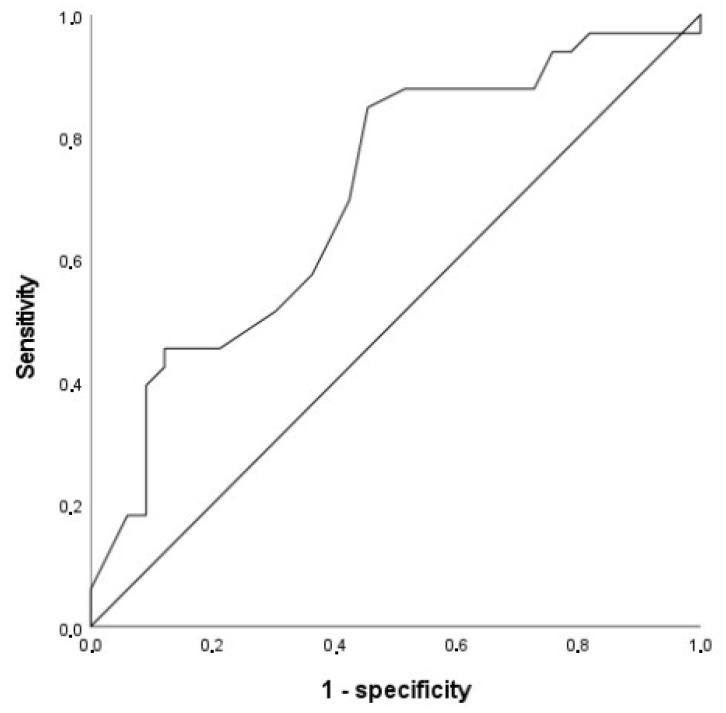
Curve ROC showing sensitivity and specificity of Stroop’s interference condition scores.

ROC analysis was performed using Stroop’s interference condition scores and SCD as a positive success criterion. This analysis resulted in an area under the curve (AUC) of 0.706, 95% CI [0.580–0.831], *p* = 0.004; this value means that the model has a fair diagnostic accuracy. Figure 1 illustrates the curve relating sensitivity to the 1-specificity. According to Youden’s index, an ideal cut-off = 49 in the Stroop’s interference condition (naming word’s colors) was determined, corresponding to a sensitivity of 0.848 and a 1-specificity of 0.455.

## 4. Discussion

The results obtained in the present study confirm the existence of subtle neuropsychological deficits in patients with SCD compared to a control group matched in age, biological sex, years of education, cognitive reserve, and general cognitive status. Regarding neuropsychological assessment, SCD showed lower performance than the control group in the three cognitive domains explored: episodic memory (delayed recall), executive functions (inhibitory control), and language (comprehension of sentences not fitted to canonical word order in Spanish), as expected by the hypothesis.

Logistic regression results revealed that the only variable that contributed significantly to participants’ classification was their performance in the Stroop’s interference condition. This result is compatible with our last hypothesis. ROC analysis provided an ideal cut-off value in this dependent variable that has a relatively high sensitivity (being good at identifying true positives, or cases with SCD), and medium specificity (and good enough at identifying true negatives). Furthermore, the observed area under the curve was greater than 0.5, which means that the model is performing better than random chance. A cut-off equal to 49 in the Stroop’s interference condition was suggested for clinical purposes.

Recent research has found slight but significant neuropsychological differences between SCD and control older adults, thus serving as evidence in line with our results. For example, in a study conducted with a total of 135 participants, including 23 healthy older adults and 30 SCD-plus (as well as 45 amnestic MCI and 37 AD patients), Hao et al. [35] reported that scores corresponding to the immediate and delayed recall of an auditory verbal learning test (World Health Organization-University of California, Los Angeles, Auditory Verbal Learning Test [AVLT]) were sensitive indicators of the cognitive decline and impairment in SCD-plus in contrast to normal controls. A recent study [20] indicated that, despite clinically unimpaired cognitive performance, SCD is linked to slight deficits in memory, executive function, and language skills. The authors discovered a correlation between these subtle cognitive deficits and cerebrospinal fluid biomarkers for AD. They concluded that these minor differences could be valid indicators and may have potential utility for the early detection of underlying preclinical AD.

More recently, Jessen et al. [36] presented findings from both cross-sectional and longitudinal data collected from 2014 to 2018 in the DELCODE study, which involved participants aged 60 and older. In this study, the group with SCD (*n* = 445) exhibited slightly poorer cognitive performance, along with more subtle functional and behavioral symptoms, compared to the control group (*n* = 236). At baseline, among various results highlighting significant differences between the groups, the SCD group demonstrated lower factor scores in memory, language abilities, and executive function than the control group. These scores were derived from a confirmatory factor analysis of individual test data across all assessments in the DELCODE neuropsychological test battery. The SCD–A+ cases (39.3% of all SCD), those in the AD continuum at stage 2 as indicated by positive amyloid biomarkers, showed greater hippocampal atrophy, lower cognitive and functional performance, and more behavioral symptoms than matched control participants. Amyloid concentration in the CSF had a greater effect on longitudinal cognitive decline in SCD than in the control group. Morrison and Oliver [37] followed up annually 3019 normal older adults for a maximum of 15 years, including 831 SCD participants. Their results pointed out that people with SCD exhibited lower baseline scores and a steeper decline in global cognition, episodic memory, semantic memory, and perceptual speed. This group (SCD) did not differ from older adults without SCD in baseline visuospatial ability or working memory but exhibited increased change over time in those two domains in contrast to them. The authors suggested that older adults with SCD may be aware of subtle cognitive declines that occur over time in all those previously mentioned domains.

Regarding the relevant role of inhibitory control (Stroop’s interference condition scores) in participants’ classification, although studies that are related to this issue are scarce, they found results that would highlight the importance of inhibitory control when distinguishing subjects with SCD from control subjects. For instance, a study conducted with older adults with SCD (*n* = 35) and cognitively intact controls (*n* = 31) [24] reported that groups differ in Stroop’s inhibitory control, and this difference is relevant in distinguishing cognitive performance between individuals with SCD and healthy controls. Seo et al. [38] explored the neuropsychological characteristics in a group of pre-MCI (*n* = 77) and cognitively normal older adults (*n* = 188) to provide measures sensitive to cognitive change in pre-MCI. They found that the pre-MCI group showed significantly lower scores in visual immediate recall, fluency tests, and the Stroop’s interference condition than those obtained by the control group. Ebenau et al. [39] classified 693 participants with SCD (60 ± 9 years) according to the ATN classification system (amyloid, tau, neurodegeneration); a follow-up (3 ± 2 years) was performed for 342 participants. As a control population, they included 124 participants without SCD. All underwent extensive neuropsychological assessment. They observed, among many other interesting results, a steeper cognitive decline in SCD with an ATN profile on TMT-A, TMT-B, and Stroop’s interference condition. However, in the control group, they found no significant associations between ATN profiles and (cross-sectional or longitudinal) cognitive test scores.

Stroop-type interference is related to the response inhibition process, which, in turn, is part of the inhibitory control mechanism [40]. There is an open debate concerning the existence of a general inhibition deficit in older adults [41]. According to this, older adults are less able to suppress dominant and well-learned responses and/or to ignore irrelevant information than younger adults are. In the classic Stroop test, participants are asked to name the color of the ink in which a word is printed (effortful response), rather than reading the word itself, which is the automatic response to suppress in an adult reader. It seems that at least some components of inhibitory control are compromised in old age, and they may severely compromise individuals’ independence and quality of life [42,43]. This decline in inhibitory control can be attributed to several factors. One possibility is that as we age, the brain undergoes structural and functional changes that affect areas responsible for executive functions [24], such as the prefrontal cortex. This should be augmented in SCD individuals, given the subtle deficits they have in higher executive load conditions [44]. Additionally, older adults may have accumulated a lifetime of knowledge and experiences, which can lead to stronger automatic responses that are harder to suppress [45]. As inhibitory control weakens, as in SCD, older adults may find it more challenging to focus on relevant stimuli, which can impact their daily functioning and overall cognitive performance. Impulse decontrol in people at risk of dementia is characterized by irritability, agitation, and rigidity [46]. For example, different background noises affect speech recognition and self-rated listening effort differently depending on age, hearing ability, and individuals’ working memory capacity and inhibitory control [47]. Strong responsiveness to food and insufficient inhibitory control are thought to be implicated in the development and maintenance of obesity; as a result, there are some studies focused on how far inhibitory control is a promising target for weight loss interventions [48]. However, it is also important to note that while some components of inhibitory control may decline, older adults often compensate for these changes with accumulated knowledge and experience, which can enhance their performance in certain contexts.

The present study has three main limitations: the sample size in each group and its cross-sectional design, which prevents determining the exact proportion of the SCD group that may progress to AD. An additional limitation is that only older adults were studied, and all of them were European Spanish-speaking individuals. Another limitation is that it was not a community-based study, as participants were mobile and able to attend centers and associations. It is also important to note that any relationship between SCD and objective cognition should be more difficult to detect cross-sectionally than longitudinally. Future research should focus on refining SCD selection criteria to better study the progression of individuals at risk for AD.

In conclusion, this study highlights subtle but significant neuropsychological differences in older adults with subjective cognitive decline (SCD) compared to a matched control group, specifically in delayed recall, inhibitory control, and sentence comprehension. Additionally, Stroop’s interference condition emerged as a key discriminator for participants’ classification, with a proposed clinical cut-off score offering potential utility for early detection. These results contribute to a growing body of evidence supporting the need for comprehensive cognitive assessment to better understand and monitor SCD in aging populations.

## Figures and Tables

**Table 1:** Mean and standard deviation (SD) and multivariate ANOVA results for participants’ socio-demographic variables and all dependent variables across cognitive domains.

	GROUP		*F* (1,63)=	Significance *p*=	Partial Eta Square	Observed Power
	Controls *Mean (SD)*	SCD *Mean (SD)*				
Age	70.39 (4.31)	70.30 (4.33)	0.00	0.96	0.000	0.050
Years of education	14.58 (5.64)	14.85 (5.31)	0.09	0.75	0.002	0.061
Cognitive reserve	15.30 (3.59)	15.18 (3.24)	0.01	0.92	0.000	0.051
MMSE	29.13 (1.10)	28.67 (1.19)	2.07	0.15	0.032	0.294
MEMORY						
Digit reordering	12.75 (1.80)	12.00 (2.16)	2.38	0.13	0.036	0.331
Digits: backward	5.63 (1.79)	5.87 (2.31)	0.21	0.65	0.003	0.074
Word List—immediate recall	31.30 (5.56)	29.58 (6.86)	0.11	0.74	0.002	0.063
Word List—delayed recall	7.94 (2.39)	5.94 (2.97)	9.06	0.004	0.126	0.843
EXECUTIVE FUNCTIONS						
FAS	41.03 (14.08)	39.79 (12.84)	0.14	0.71	0.002	0.066
Semantic Verbal Fluency (animals + fruits)	33.55 (6.57)	30.94 (6.62)	2.28	0.14	0.035	0.318
TMT-A time	50.94 (16.64)	54.36 (17.44)	0.85	0.36	0.013	0.148
Stroop’s interference condition	49.34 (7.99)	43.13 (8.69)	9.50	0.003	0.131	0.859
LANGUAGE						
BNT	53.44 (4.72)	52.44 (5.78)	1.32	0.25	0.021	0.205
ECCO: non-canonical sentences	14.72 (1.98)	13.57 (2.25)	4.02	0.049	0.060	0.506
ECCO: sentences with 2 propositions	15.69 (2.24)	14.57 (2.55)	2.93	0.09	0.044	0.392

Notes. MMSE: Mini-Mental State Exam; FAS: Phonemic Verbal Fluency with the letters F, A, and S; TMT-A: Trail-Making Test form A; BNT: Boston Naming Test; ECCO: Exploración Cognitiva de la Comprensión de Oraciones (English trad.: Cognitive assessment of sentence comprehension).

**Table 2:** Summary of logistic regression final equation.

	B	Standard Error	Wald	df	Sig.	Exp(B)
Delayed recall	−0.200	0.112	3.22	1	0.073	0.818
Stroop’s interference	−0.090	0.033	7.35	1	0.007	0.914
ECCO non-canonical sentences	0.025	0.162	0.026	1	0.873	1.026

## Data Availability

The data that support the findings of this study are available from the first author (R.L.-H.).

## References

[1] Jessen F., Amariglio R.E., Van Boxtel M., Breteler M., Ceccaldi M., Chételat G., Dubois B., Dufouil C., Ellis K.A., Van Der Flier W.M. (2014). Subjective Cognitive Decline Initiative. A conceptual framework for research on subjective cognitive decline in preclinical Alzheimer’s disease. Alzheimers Dement..

[2] Petersen R.C., Caracciolo B., Brayne C., Gauthier S., Jelic V., Fratiglioni L. (2014). Mild cognitive impairment: A concept in evolution. J. Intern. Med..

[3] Jessen F., Amariglio R.E., Buckley R.F., van der Flier W.M., Han Y., Molinuevo J.L., Rabin L., Rentz D.M., Rodriguez-Gomez O., Saykin A.J. (2020). The characterization of subjective cognitive decline. Lancet Neurol..

[4] Chen S.T., Siddarth P., Ercoli L.M., Merrill D.A., Torres-Gil F., Small G.W. (2014). Modifiable risk factors for Alzheimer’s disease and subjective memory impairment across age groups. PLoS ONE.

[5] Rabin L.A., Smart C.M., Crane P.K., Amariglio R.E., Berman L.M., Boada M., Buckley R.F., Chételat G., Dubois B., Ellis K.A. (2015). Subjective cognitive decline in older adults: An overview of self-report measures used across 19 international research studies. J. Alzheimer’s Dis..

[6] Cheng Y.W., Chen T.F., Chiu M.J. (2017). From mild cognitive impairment to subjective cognitive decline: Conceptual and methodological evolution. Neuropsychiatr. Dis. Treat..

[7] Mitchell A.J., Beaumont H., Ferguson D., Yadegarfar M., Stubbs B. (2014). Risk of dementia and mild cognitive impairment in older people with subjective memory complaints: Meta-analysis. Acta Psychiatr. Scand..

[8] Rotenberg S., Maeir A., Dawson D.R. (2020). Changes in Activity Participation Among Older Adults with Subjective Cognitive Decline or Objective Cognitive Deficits. Front. Neurol..

[9] Li H., Tan C.C., Tan L., Xu W. (2023). Predictors of cognitive deterioration in subjective cognitive decline: Evidence from longitudinal studies and implications for SCD-plus criteria. J. Neurol. Neurosurg. Psychiatry.

[10] Roehr S., Villringer A., Angermeyer M.C., Luck T., Riedel-Heller S.G. (2016). Outcomes of stable and unstable patterns of subjective cognitive decline—Results from the Leipzig Longitudinal Study of the Aged (LEILA75+). BMC Geriatr..

[11] Yu H.H., Tan C.C., Huang S.J., Zhang X.H., Tan L., Xu W., Alzheimer’s Disease Neuroimaging Initiative (2024). Predicting the reversion from mild cognitive impairment to normal cognition based on magnetic resonance imaging, clinical, and neuropsychological examinations. J. Affect. Disord..

[12] Jonker C., Geerlings M.I., Schmand B. (2000). Are memory complaints predictive for dementia? A review of clinical and population-based studies. Int. J. Geriatr. Psychiatry.

[13] Garcia-Ptacek S., Cavallin L., Kåreholt I., Kramberger M.G., Winblad B., Jelic V., Eriksdotter M. (2014). Subjective cognitive impairment subjects in our clinical practice. Dement. Geriatr. Cogn. Disord. Extra.

[14] Rijs K.J., Van den Kommer T.N., Comijs H.C., Deeg D.J. (2015). Prevalence and incidence of memory complaints in employed compared to non-employed aged 55–64 years and the role of employment characteristics. PLoS ONE.

[15] Zhou C., Jeryous Fares B., Thériault K., Trinh B., Joseph M., Jauhal T., Sheppard C., Labelle P.R., Krishnan A., Rabin L. (2024). Subjective cognitive decline and objective cognitive performance in older adults: A systematic review of longitudinal and cross-sectional studies. J. Neuropsychol..

[16] Costa A.N., Nowakowski L.M., McCrae C.S., Cowan N., Curtis A.F. (2023). Discrepancies in Objective and Subjective Cognition in Middle-Aged and Older Adults: Does Personality Matter?. Gerontol. Geriatr. Med..

[17] Nuzum H., Dorociak K., Kamil-Rosenberg S., Louras P., Mostofi M., Fairchild J.K. (2020). Objective and Subjective Cognitive Function, and Relations with Quality of Life and Psychological Distress. Innov. Aging.

[18] Balash Y., Mordechovich M., Shabtai H., Giladi N., Gurevich T., Korczyn A.D. (2013). Subjective memory complaints in elders: Depression, anxiety, or cognitive decline?. Acta Neurol. Scand..

[19] Mulligan B.P., Smart C.M., Ali J.I. (2016). Relationship of subjective and objective performance indicators in subjective cognitive decline. Psychol. Neurosci..

[20] Wolfsgruber S., Kleineidam L., Guski J., Polcher A., Frommann I., Roeske S., Spruth E.J., Franke C., Priller J., Kilimann I. (2020). DELCODE Study Group. Minor neuropsychological deficits in patients with subjective cognitive decline. Neurology.

[21] Zheng Z., Zhao X., Cui X., Liu X., Zhu X., Jiang Y., Li J. (2023). Subtle Pathophysiological Changes in Working Memory-Related Potentials and Intrinsic Theta Power in Community-Dwelling Older Adults with Subjective Cognitive Decline. Innov. Aging.

[22] Hong Y.J., Park J.W., Lee S.B., Kim S.H., Kim Y., Ryu D.W., Park K.W., Yang D.W. (2022). The influence of amyloid burden on cognitive decline over 2 years in older adults with subjective cognitive decline: A prospective cohort study. Dement. Geriatr. Cogn. Disord..

[23] Corlier F.W., Shaw C., Hayes-Larson E., Mungas D., Farias S.T., Glymour M.M., Whitmer M.A., Mayeda E.R. (2020). Association Between Cognitive Test Performance and Subjective Cognitive Decline in a Diverse Cohort of Older Adults: Findings from the KHANDLE Study. Alzheimer Dis. Assoc. Disord..

[24] López-Higes R., Prados J.M., Rubio S., Montejo P., Del Río D. (2017). Executive functions and linguistic performance in SCD older adults and healthy controls. Aging Neuropsychol. Cogn..

[25] López-Higes R., Rubio-Valdehita S., Fernandes S.M., Rodrigues P.F. (2024). Differentiation between Normal Cognition and Subjective Cognitive Decline in Older Adults Using Discrepancy Scores Derived from Neuropsychological Tests. Geriatrics.

[26] Lobo A., Saz P., Marcos G., Día J.L., De La Cámara C., Ventura T., Morales Asín F., Fernando Pascual L., Montañés J.A., Aznar S. (1999). Revalidation and standardization of the cognition mini-exam (first Spanish version of the Mini-Mental Status Examination) in the general geriatric population. Med. Clínica.

[27] Sheikh J., Yesavage J., Brink T. (1986). Geriatric Depression Scale (GDS): Recent findings and development of a shorter version. Clinical Gerontology: A Guide to Assessment and Intervention.

[28] Wechsler D. (2004). WMS-III Escala de Memoria de Wechsler-III.

[29] Rami L., Valls-Pedret C., Bartres-Faz D., Caprile C., Sole-Padulles C., Castellvi M., Olives B., Bosch J.L., Molinuevo J.L. (2011). Cognitive Reserve Questionnaire. Values obtained in a healthy elderly population and in Alzheimer’s disease. Rev. De Neurol..

[30] MacDonald M.C., Almor A., Henderson V.W., Kempler D., Andersen E.S. (2001). Assessing working memory and language comprehension in Alzheimer’s disease. Brain Lang..

[31] Golden C.J. (1978). Stroop Color and Word Test: A Manual for Clinical and Experimental Uses.

[32] Reitan R.M. (1992). Trail Making Test: Manual for Administration and Scoring.

[33] García-Albea J.E., Sánchez-Bernardos M.L., del Viso-Pabón S., Goodglass H., Kaplan E. (1986). Boston Naming Test for Aphasia Diagnosis: Spanish version. Assessment of Aphasia and Related Disorders.

[34] López-Higes R., Rubio S., Martín-Aragoneses M.T., Del Río D., Mejuto G. (2012). Assessment of grammatical comprehension in normal and pathological aging: A summary of the results obtained with ECCO and ECCO_Senior tests. Int. J. Psychol. Res..

[35] Hao L., Xing Y., Li X., Mu B., Zhao W., Wang G., Wang T., Jia J., Han Y. (2019). Risk factors and neuropsychological assessments of subjective cognitive decline (plus) in Chinese memory clinic. Front. Neurosci..

[36] Jessen F., Jessen F., Wolfsgruber S., Kleineindam L., Spottke A., Altenstein S., Bartels C., Berger M., Brosseron F., Daamen M. (2023). Subjective cognitive decline and stage 2 of Alzheimer’s disease in patients from memory centers. Alzheimer’s Dement..

[37] Morrison C., Oliver M.D. (2023). Subjective cognitive decline is associated with lower baseline cognition and increased rate of cognitive decline. J. Gerontol. Ser. B.

[38] Seo E.H., Kim H., Lee K.H., Choo I.L.H. (2016). Altered executive function in pre-mild cognitive impairment. J. Alzheimer’s Dis..

[39] Ebenau J.L., Timmers T., Wesselman L.M., Verberk I.M., Verfaillie S.C., Slot R.E., van Harten A.C., Teunissen C.E., Barkhof F., Bosch K.A.v.D. (2020). ATN classification and clinical progression in subjective cognitive decline: The SCIENCe project. Neurology.

[40] Diamond A. (2020). Executive Functions. Handbook of Clinical Neurology.

[41] Rey-Mermet A., Gade M. (2018). Inhibition in Aging: What Is Preserved? What Declines? A Meta-Analysis. Psychon. Bull. Rev..

[42] Forte G., Troisi G., Favieri F., Casagrande M. (2024). Inhibition changes across the lifespan: Experimental evidence from the Stroop task. BMC Psychol..

[43] Campbell K.L., Lustig C., Hasher L. (2020). Aging and inhibition: Introduction to the special issue. Psychol. Aging.

[44] Macoir J., Tremblay P., Hudon C. (2022). The use of executive fluency tasks to detect cognitive impairment in individuals with subjective cognitive decline. Behav. Sci..

[45] Troyer A.K., Leach L., Strauss E. (2006). Aging and response inhibition: Normative data for the Victoria Stroop Test. Aging Neuropsychol. Cogn..

[46] Saari T., Smith E.E., Ismail Z. (2022). Network analysis of impulse dyscontrol in mild cognitive impairment and subjective cognitive decline. Int. Psychogeriatr..

[47] Stenbäck V., Marsja E., Hällgren M., Lyxell B., Larsby B. (2021). The contribution of age, working memory capacity, and inhibitory control on speech recognition in noise in young and older adult listeners. J. Speech Lang. Hear. Res..

[48] De Klerk M.T., Smeets P.A.M., La Fleur S.E. (2023). Inhibitory control as a potential treatment target for obesity. Nutr. Neurosci..

